# Neutrophil-to-Lymphocyte Ratio as a Predictor of Mortality and Clinical Outcomes in Heart Failure Patients

**DOI:** 10.7759/cureus.83359

**Published:** 2025-05-02

**Authors:** Anurag Rawat, Kinnari Vyas

**Affiliations:** 1 Department of Interventional Cardiology, Himalayan Institute of Medical Sciences, Dehradun, IND; 2 Department of Plastic Surgery, Shri Guru Ram Rai Institute of Medical and Health Sciences, Dehradun, IND

**Keywords:** diagnostic predictor, heart failure, lymphocyte, mortality, neutrophil, predictor

## Abstract

The neutrophil-to-lymphocyte ratio (NLR) is an emerging biomarker reflecting systemic inflammation, playing a critical role in heart failure (HF) prognosis. Elevated NLR, indicative of neutrophilia and lymphocytopenia, correlates with worsened outcomes, including higher mortality and adverse cardiac events. Studies highlight its utility as a robust indicator for risk stratification and management in both acute and chronic HF conditions. This study aims to analyze the correlation of NLR as a predictor of mortality in acute heart failure patients. This systematic review and meta-analysis explored the relationship between the neutrophil-to-lymphocyte ratio (NLR) and clinical outcomes in heart failure (HF) patients, including mortality, rehospitalization, disease progression, and functional capacity. A comprehensive search of PubMed, Scopus, Embase, and Web of Science identified 38 studies meeting the inclusion criteria. Quality and bias were assessed using established tools, and statistical analyses evaluated pooled effect sizes, heterogeneity, and optimal NLR cutoffs, evaluating their prognostic accuracy for HF outcomes. The findings highlight that heart failure (HF) patients who survived had significantly lower neutrophil-to-lymphocyte ratio (NLR) values compared to those who died (pooled standardized mean difference {SMD} = -0.48; 95% confidence interval {CI}: -0.54, -0.43; p < 0.05). Elevated NLR is significantly associated with increased mortality in heart failure patients, with most studies showing strong inverse associations and odds ratios (ORs) below 1. Odds ratios further supported this, with higher NLR linked to increased mortality risk (e.g., OR = 0.38). The area under the curve (AUC) of 0.834 indicates strong predictive accuracy for mortality, with optimal NLR cutoffs of 5.91 and 6.18 balancing sensitivity (84.6%) and specificity (84.6%). High heterogeneity (I² = 90%) shows the variability among studies. The study has concluded that an elevated neutrophil-to-lymphocyte ratio (NLR) is consistently associated with increased mortality in patients with heart failure (HF). Thus, the neutrophil-to-lymphocyte ratio (NLR) can be used as a reliable prognostic marker in patients with heart failure.

## Introduction and background

**Figure 1:**
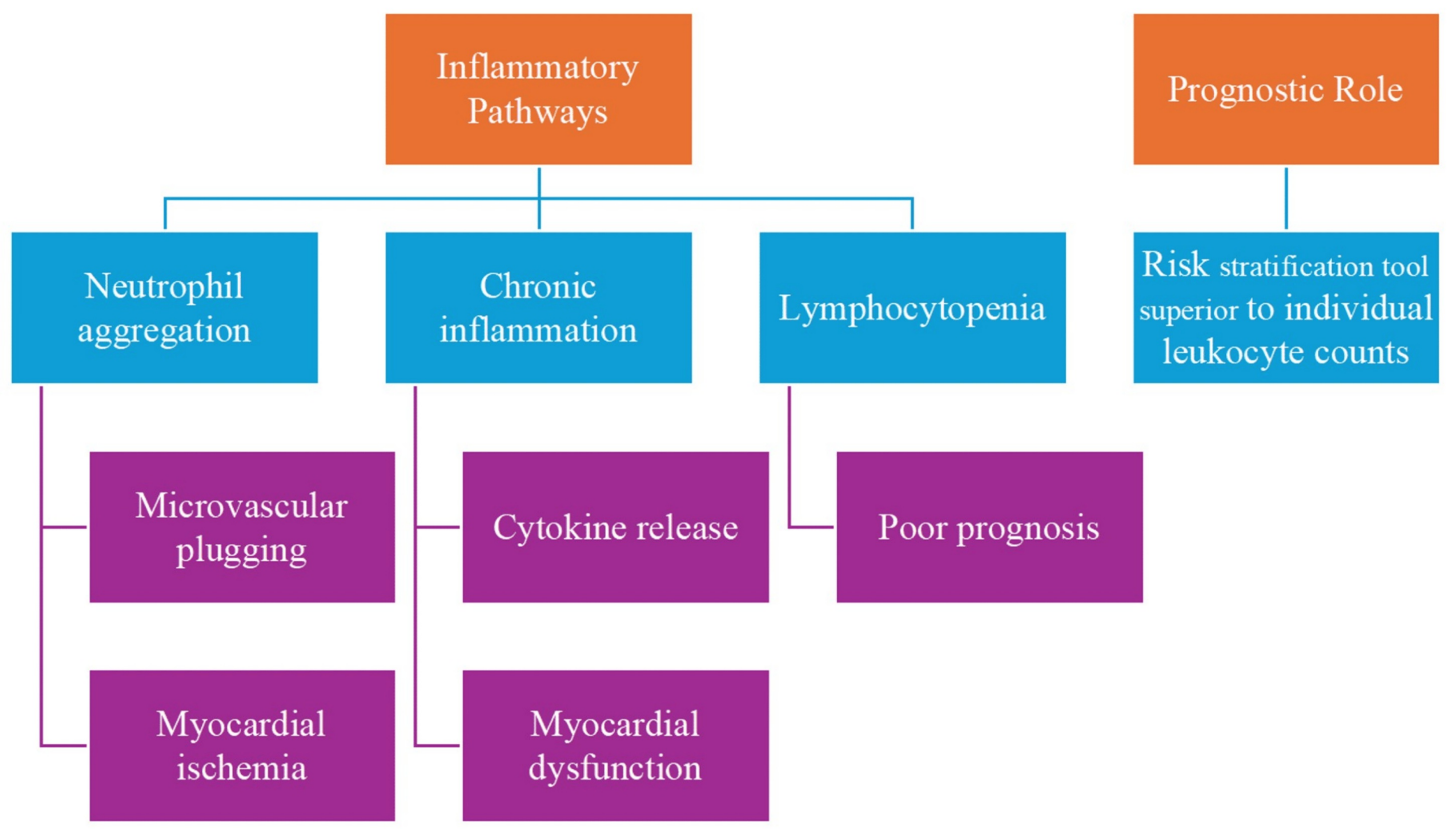
Mechanisms of elevated NLR in HF Image credits: Anurag Rawat and Kinnari Vyas NLR, neutrophil-to-lymphocyte ratio; HF, heart failure

The neutrophil-to-lymphocyte ratio (NLR) is a crucial biomarker that provides insight into the inflammatory status of patients with heart failure (HF) [1]. NLR represents the balance between the innate and adaptive immune systems, thus acting as a useful indicator of the overall inflammatory response. Neutrophils are representative of the acute phase of the inflammatory response, while lymphocytes are involved in the adaptive immune system and have been linked to chronic inflammation. An elevated NLR indicates an imbalance favoring the innate immune response over the adaptive immune system. Such an elevated NLR signifies an intensified inflammatory state. Several investigations have proven that the NLR in HF patients has clinical relevance as a prognostic factor. Yost et al. reported the occurrence of postoperative right ventricular failure and lower survival probability associated with elevated NLR values before left ventricular assist device (LVAD) implantation [2]. The mechanisms involved may underline the relationship between the NLR and inflammation in HF. Neutrophils may aggregate with platelets, leading to microvascular plugging and myocardial ischemia. Additionally, lymphocytopenia is associated with increased mortality and poor prognosis in advanced HF patients. These findings indicate that the NLR provides a comprehensive assessment of the inflammatory condition, which is critical in the pathology and progression of HF [1,2]. Similarly, Yan et al. reported that elevated NLR was associated with adverse events in elderly patients with chronic HF, after adjusting for various clinical factors [3]. The NLR has been known to predict the outcome for patients with acute HF as well. In their findings, Cho et al. concluded that individuals with a higher NLR had higher readmission at 30 days and high mortality in the long term than those who had a low NLR. High NLR also leads to the risk of increased mortality or heart transplantation (HTx) in patients with advanced HF [4]. The current evidence indicates an increasing role of NLR in the context of predictors for mortality and unfavorable results from cardiovascular diseases (Figure 1).

Elevated neutrophil-to-lymphocyte ratio in heart failure reflects increased inflammation, through neutrophils causing microvascular plugging and ischemia and lymphopenia indicating poor adaptive immunity and increased mortality. Increased chronic inflammation further worsens myocardial dysfunction through cytokine activation [4]. It is during the progression of HF that the imbalance between the innate and adaptive immune system becomes more intense, at which point NLR becomes a better biomarker for systemic inflammation. High NLR can predict hospitalization, HF progression, and mortality; it is therefore an essential risk stratification and management tool. The literature already confirms the prognostic role of NLR in predicting clinical outcome in a wide scope of cardiovascular conditions. So, Larmann et al. proved that major adverse cardiovascular and cerebrovascular events during non-cardiac surgeries after coronary heart disease correspond to elevated preoperative NLRs [5]. This is in line with previous reports suggesting that NLR can be a stronger independent predictor of mortality than other leukocyte subpopulations after myocardial infarction [5-7]. The utility of NLR extends beyond coronary artery disease. Argan et al. [6] reported that an increased NLR was associated with worsening renal function in patients with chronic heart failure [8-10]. Additionally, Haj-Yehia et al. [7] observed that a high NLR was linked to cancer therapy-related cardiovascular toxicity in high-risk cancer patients receiving immune checkpoint inhibitor therapy [11-14]. Furthermore, evidence suggests that NLR is a better predictor of mortality than independent absolute neutrophil and lymphocyte counts in patients with advanced heart failure. Benites-Zapata et al. [8] observed NLR to be a greater mortality predictor than neutrophilia or lymphopenia in these patients [15]. The value of NLR in terms of prognosis is not confined only to cardiovascular diseases. In patients with chronic kidney disease, Zhao et al. presented a systematic review and meta-analysis where an elevated NLR was associated with an increased risk of all-cause mortality and cardiovascular events [9]. This points out the utility of NLR as a general marker of inflammation that will be useful for prognostic information in different states of diseases [9].

The elevation of NLR is considered an indicator of inflammation, with high values showing poor outcomes for patients suffering from both acute and chronic HF [16]. Neutrophils aggregating with platelets block microvascular sites, leading to myocardial ischemia; lymphopenia represents blunted adaptive immunity, thus the increase in mortality. In addition to inflammation mediators, C-reactive protein and interleukin-6 increase the progression of HF [17,18]. Many studies have shown that NLR is a powerful prognostic marker, even superior to independent leukocyte counts in the prediction of adverse events, including hospitalization, the progression of HF, and mortality. Thus, NLR can be applied in clinical risk stratification and management [19].

Despite the compelling evidence linking elevated neutrophil-to-lymphocyte ratio (NLR) with increased mortality in heart failure patients, several research gaps remain. Notably, there is limited exploration of how this association varies across different heart failure subtypes, clinical settings, and demographic groups such as age, sex, and ethnicity. Many studies employ heterogeneous cutoff values and lack standardized thresholds, making it challenging to generalize findings or establish universal clinical guidelines. Additionally, the absence of longitudinal data restricts the understanding of how dynamic changes in NLR over time may influence patient outcomes. The underlying pathophysiological mechanisms connecting elevated NLR to adverse events in heart failure also remain insufficiently elucidated. Further prospective, multicenter studies with standardized protocols are necessary to validate NLR as a reliable and universally applicable prognostic marker in diverse clinical contexts.

## Review

Method

Research Design

This systematic review and meta-analysis analyzed the relationship between the neutrophil-to-lymphocyte ratio (NLR) and clinical outcomes in heart failure (HF) patients, considering variations in study design, including cross-sectional, prospective cohort and retrospective cohort studies. Each design's methodological differences were factored into the analysis to account for their unique contributions to understanding NLR's prognostic value. Following the Population, Exposure, Comparator, Outcome, and Study (PECOS) framework, the study focused on HF patients as the population. It examined NLR's impact on key outcomes such as mortality, rehospitalization, HF prediction, extended hospital stay, and progression to renal disease. Peer-reviewed studies in English with cross-sectional, case-control, cohort, or randomized clinical trial designs were included, while case reports, editorials, and animal studies were excluded. The study utilized rigorous search strategies across various online databases up to July 2024. Data extraction and quality assessments followed standardized methodologies with tools such as Microsoft Excel (Microsoft Corp., Redmond, WA).

Inclusion and Exclusion Criteria

Inclusion: This study included only peer-reviewed studies published in English and available in full-text format. The selected studies focused on the participants diagnosed with heart failure (HF) and specifically evaluated the relationship between the neutrophil-to-lymphocyte ratio (NLR) and clinical outcomes in HF patients. Various study designs were considered, including cross-sectional, case-control, cohort studies and randomized clinical trials (RCTs). Additionally, only those studies that provided sufficient data on at least one of the desired outcomes, mortality, rehospitalization, HF prediction, pulmonary vascular resistance, and atrial fibrillation (AF), were included in the analysis.

Exclusion: This study excluded meeting abstracts, editorials, case reports, and case series papers. Additionally, studies with incomplete or insufficient data on the desired outcomes were not included. Furthermore, research conducted on animal models was excluded to ensure that the analysis was based solely on human studies.

Strategy of Literature Search

This search strategy provided a comprehensive collection of relevant records for this systematic review. This study used electronic medical databases to collect accurate and relevant papers, which helped complete this study. This study used PubMed, Scopus, Embase, China National Knowledge Infrastructure (CNKI), Cochrane, and Web of Science, which covered publications up to July 2024. Furthermore, this study used Medical Subject Headings (MeSH) and non-MeSH terms across all databases. For the neutrophil-to-lymphocyte ratio (NLR), which included ("neutrophil* to lymphocyte* ratio" OR "neutrophil -lymphocyte" OR "neutrophil*-lymphocyte* ratio" OR "neutrophil* to lymphocyte*" OR "neutrophil*-to-lymphocyte* ratio" OR "neutrophil*-to lymphocyte* ratio" OR "neutrophil* to-lymphocyte* ratio" OR "neutrophil /lymphocyte ratio" OR "neutrophil*/lymphocyte*" OR "nlr"). Again, for heart failure (HF), it included ("heart failure" OR "cardiac failure" OR "heart insufficiency" OR "cardiac insufficiency" OR "congestive heart failure" OR "congestive cardiac failure" OR "decompensated heart failure" OR "decompensated cardiac failure" OR "decompensated heart insufficiency" OR "decompensated cardiac insufficiency" OR "acute decompensated heart failure" OR "acute decompensated cardiac failure" OR "acute decompensated heart insufficiency" OR "acute decompensated cardiac insufficiency" OR "hf").

Selection Process

This study considered those papers that had full texts of all relevant articles, which were then reviewed. In cases of duplicate articles, only one version was included. Disagreements during the screening process were resolved through discussion between reviewers, and if consensus was not reached, a third reviewer was consulted.

**Figure 2:**
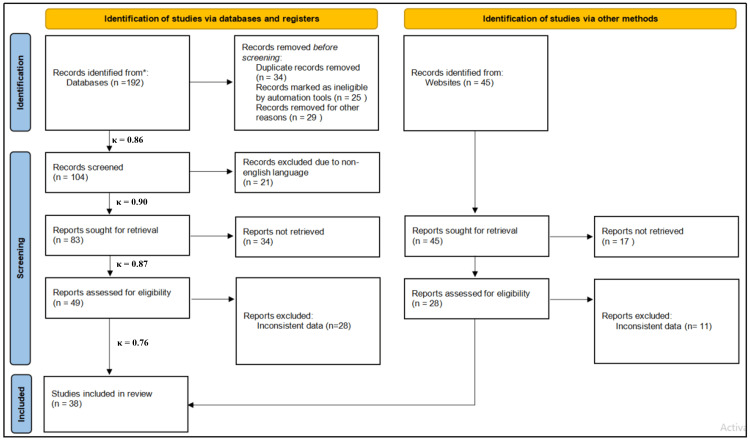
PRISMA flowchart PRISMA: Preferred Reporting Items for Systematic Reviews and Meta-Analyses *PubMed, Scopus, Embase, China National Knowledge Infrastructure (CNKI), Cochrane, and Web of Science

The Preferred Reporting Items for Systematic Reviews and Meta-Analyses (PRISMA) flowchart (Figure 2) has shown the study selection process for a systematic review, showing how studies were identified, screened, assessed for eligibility, and included in the final analysis. During the identification phase, 192 records were retrieved from databases and registers, while an additional 45 records were identified through other methods, such as websites. Of the database records, 34 were removed as duplicates, 25 were deemed ineligible by automation tools, and 29 were excluded for other reasons. This left 104 records to be screened. In this screening step, 21 records were excluded for being in a non-English language (due to language barrier), leaving 83 reports to be retrieved. However, 34 reports could not be retrieved, resulting in 49 reports being assessed for eligibility. Of these, 28 were excluded due to inconsistent data, leaving a total of 38 studies from databases included in the review.

Similarly, for the records identified via other methods, all 45 records were sought for retrieval, but 17 could not be retrieved. This left 28 reports to be assessed for eligibility, of which 11 were excluded due to inconsistent data. In summary, the flowchart demonstrates a thorough and systematic process of study selection, resulting in a total of 38 studies being included in the review.

Data Gathering Process

This study included the first author's name and publication year, sample size, the frequency of male participants, and age or median with interquartile range (IQR), as available. On the other hand, the additional information included the follow-up period, neutrophil-to-lymphocyte ratio (NLR) values, tertiles, quartiles, and heart failure (HF) outcomes. These results encompass mortality, rehospitalization, HF prediction, pulmonary vascular resistance, atrial fibrillation (AF), progression to renal disorder, and functional capacity.

Risk of Bias Assessment

The risk of bias assessment was conducted in MetaXL software (EpiGear International Pty Ltd., Sunrise Beach, Australia), which comes as a plugin of Microsoft Excel. This evaluated the quality and risk of bias in each included study. For cross-sectional studies, Microsoft Excel was applied, while case-control studies were evaluated using the National Institutes of Health (NIH) quality assessment tool. Cohort studies were evaluated with the Joanna Briggs Institute (JBI) critical appraisal checklist for cohort studies, and randomized clinical trials (RCTs) were assessed using the JBI critical appraisal checklist for RCTs. Also, the certainty of predefined outcomes was analyzed using the Grading of Recommendations Assessment, Development and Evaluation (GRADE) framework. Inter-reviewer reliability was assessed using Cohen's kappa statistics during each screening and eligibility stage, yielding values between 0.76 and 0.90, which indicate substantial to almost perfect agreement. A random-effects model was employed for all meta-analyses. The random-effects approach assumes that the true effects vary across studies and provides a more conservative estimate by incorporating both within-study and between-study variance.

Statistical Analysis

The pooled effect sizes were presented as mean values and hazard ratios (HR) with 95% confidence intervals (CI), as applicable. For continuous variables, medians with interquartile ranges (IQR) or ranges were converted to mean ± standard deviation (SD). Heterogeneity was evaluated by using Cochran's Q statistic, I², and tau squared (τ²), and a random-effects model was used for all downstream analyses. The discriminatory power of NLR was evaluated using receiver operating characteristic (ROC) curve analysis, and optimal cutoff points were identified using Youden's index to balance sensitivity and specificity. Area under the curve (AUC) values were calculated to determine the accuracy of NLR as a prognostic marker. Beyond the I² statistic, heterogeneity was also evaluated using Cochran's Q and tau squared (τ²). Additionally, potential bias across individual studies was addressed using funnel plot symmetry and Egger's regression test to assess publication bias. Sensitivity analyses and subgroup analyses were performed to explore the sources of heterogeneity.

Result

Table 1 presents a comparative analysis of the demographic and methodological characteristics of studies investigating the role of the neutrophil-to-lymphocyte ratio (NLR) in heart failure (HF) patients. The studies encompass a mix of cross-sectional and cohort designs, highlighting variations in how NLR was evaluated across different populations. Studies showed that mortality is consistently associated with higher mean ages, aligning with the known increased mortality risk in older HF populations. Overall, the heterogeneity in design, sample size, gender distribution, and age highlights the need for the careful interpretation of pooled data, as these factors likely impact the generalizability of conclusions about NLR as a prognostic marker.

**Table 1:** Basic characteristics of the included studies SD, standard deviation; N/A, not available

Authors	Design	Total Sample Size	Survived Sample Size	Death Sample Size	Male (%) Total	Male (%) Survived	Male (%) Death	Age (Total)	Age (Survived) (Mean ± SD)	Age (Death) (Mean ± SD)
Wu et al., 2023 [20]	Cross-sectional	1207	667	540	689 (57.08%)	363 (54.42%)	326 (60.37%)	67.3 ± 12.5	63.3 ± 13	72.2 ± 9.9
Tamaki et al., 2023 [21]	Cross-sectional	1026	N/A	N/A	462 (45.03%)	N/A	N/A	82.33 ± 7.42	N/A	N/A
Liu et al., 2023 [22]	Cross-sectional	1169	986	183	673 (57.57%)	556 (56.39%)	117 (63.93%)	69.51 ± 13.83	68.87 ± 13.96	72.95 ± 12.64
Zhu et al., 2022 [23]	Prospective cohort	538	311	227	357 (66.36%)	228 (73.31%)	129 (56.83%)	61.07 ± 15.98	58.03 ± 15.91	65.23 ± 15.16
Wang et al., 2022 [24]	Cross-sectional	189	N/A	N/A	106 (56.08%)	N/A	N/A	67.07 ± 13.41	N/A	N/A
Maeda et al., 2022 [25]	Cross-sectional	669	N/A	N/A	398 (59.49%)	N/A	N/A	75.8 ± 11.3	N/A	N/A
Liu et al., 2022 [26]	Retrospective cohort	454	N/A	N/A	247 (54.41%)	N/A	N/A	76 ± 8	N/A	N/A
Li et al., 2022 [27]	Cross-sectional	50	13	37	30 (60.00%)	8 (61.54%)	22 (59.46%)	74.16 ± 2.94	75.77 ± 3.54	73.59 ± 2.51
Kocaoglu and Alatli, 2022 [28]	Cross-sectional	101	62	39	49 (48.51%)	30 (48.39%)	19 (48.72%)	73.15 ± 10.19	72.61 ± 10.92	74.00 ± 8.96
Davison et al., 2022 [29]	Cross-sectional	1823	N/A	N/A	1116 (61.22%)	N/A	N/A	71.40 ± 10.53	N/A	N/A
Davran et al., 2022 [30]	Cross-sectional	139	116	23	64 (46.04%)	57 (49.14%)	7 (30.43%)	69.2 ± 12.1	69.3 ± 11.5	69.2 ± 15
Delcea et al., 2021 [31]	Retrospective cohort	1299	N/A	N/A	624 (48.04%)	N/A	N/A	72.35 ± 10.45	N/A	N/A
Curran et al., 2021 [32]	Cross-sectional	1622	N/A	N/A	1086 (66.95%)	N/A	N/A	74 ± 10	N/A	N/A

Table 2 shows the NLR values and their associations with clinical outcomes across the studies. A consistent trend emerges: higher NLR values are observed in deceased HF patients compared to survivors. For instance, Wu et al. (2023) [20] reported a mean NLR of 2.8 in deceased patients versus 2.16 in survivors, while Liu et al. (2023) [22] found an even larger difference, with mean NLR values of 13.43 for deceased patients and 7.82 for survivors. These findings align across studies, indicating that elevated NLR strongly correlates with poorer outcomes.

**Table 2:** NLR and other findings of the included studies N/A, not available; SD, standard deviation; NLR, neutrophil-to-lymphocyte ratio

Authors	Total Sample Size	Survived Sample Size	Death Sample Size	NLR (Total) (Mean ± SD)	NLR (Survived) (Mean ± SD)	NLR (Death) (Mean ± SD)
Wu et al., 2023 [20]	1207	667	540	2.46 ± 1.18	2.16 ± 1.04	2.8 ± 1.41
Tamaki et al., 2023 [21]	1026	N/A	N/A	4.26 ± 2.74	N/A	N/A
Liu et al., 2023 [22]	1169	986	183	8.43 ± 6.21	7.82 ± 5.44	13.43 ± 12.58
Zhu et al., 2022 [23]	538	311	227	2.97 ± 1.96	2.69 ± 1.78	3.46 ± 2.29
Wang et al., 2022 [24]	189	N/A	N/A	3.46 ± 2.62	N/A	N/A
Maeda et al., 2022 [25]	669	N/A	N/A	2.62 ± 1.45	N/A	N/A
Liu et al., 2022 [26]	454	N/A	N/A	2.74 ± 1.36	N/A	N/A
Li et al., 2022 [27]	50	13	37	3.50 ± 1.78	5.12 ± 2.81	2.93 ± 0.64
Kocaoglu and Alatli, 2022 [28]	101	62	39	6.74 ± 5.44	5.54 ± 2.98	8.66 ± 7.59
Davison et al., 2022 [29]	1823	N/A	N/A	N/A	N/A	N/A
Davran et al., 2022 [30]	139	116	23	6.31 ± 4.48	5.84 ± 4.05	8.7 ± 5.79
Delcea et al., 2021 [31]	1299	N/A	N/A	3.18 ± 1.72	N/A	N/A
Curran et al., 2021 [32]	1622	N/A	N/A	Median: 3.22	N/A	N/A
Bai et al., 2021 [33]	172	N/A	N/A	3.98 ± 2.48	N/A	N/A
Arfsten et al., 2021 [34]	443	N/A	N/A	4.03 ± 2.3	N/A	N/A
Angkananard et al., 2021 [35]	321	N/A	N/A	3.7 ± 2.45	N/A	N/A
Urbanowicz et al., 2020 [36]	41	N/A	N/A	3.46 ± 1.69	N/A	N/A
Sadeghi et al., 2020 [37]	197	167	30	4.41 ± 3.64	3.84 ± 2.82	7.61 ± 5.62
Köse et al., 2020 [38]	200	162	38	4.25 ± 3.52	3.84 ± 3.28	5.98 ± 4.01
Cho et al., 2020 [10]	5580	N/A	N/A	N/A	N/A	N/A
Turcato et al., 2019 [39]	439	394	45	5.48 ± 6.61	4.43 ± 2.97	14.76 ± 16.08
Kone et al., 2019 [40]	105	81	24	2.64 ± 1.9	2.32 ± 1.07	3.75 ± 3.29
Boralkar et al., 2020 [41]	443	N/A	N/A	7.06 ± 5.57	N/A	N/A
Yurtdaş et al., 2018 [42]	40	N/A	N/A	3.2 ± 1.4	N/A	N/A
Yan et al., 2016 [3] and Yan et al., 2017 [43]	1355	422	933	3.2 ± 3.1	3.6 ± 3.1	3 ± 3
Pourafkari et al., 2017 [44]	554	N/A	N/A	6.3 ± 4.99	N/A	N/A
Huang et al., 2017 [14]	1923	1048	875	5.44 ± 6.09	4.76 ± 5.35	6.26 ± 6.8
Siniorakis et al., 2017 [45]	72	N/A	N/A	3.13 ± 2.38	N/A	N/A
Wasilewski et al., 2016 [46]	1734	N/A	N/A	2.93 ± 1.81	N/A	N/A
Liu et al., 2016 [47]	179	169	10	4.26 ± 5.45	3.9 ± 5.2	10.2 ± 6.2
Argan et al., 2016 [48]	68	N/A	N/A	2.64 ± 1.33	N/A	N/A
Fu et al., 2015 [49]	306	N/A	N/A	3.3 ± 2.23	N/A	N/A
Durmus et al., 2015 [50]	56	N/A	N/A	5.5 ± 2.8	N/A	N/A
Cakıcı et al., 2014 [51]	94	N/A	N/A	3.33 ± 3.91	N/A	N/A
Budak et al., 2015 [52]	190	N/A	N/A	6.2 ± 8.6	N/A	N/A
Benites-Zapata et al., 2015 [53]	527	N/A	N/A	4.3 ± 2.97	N/A	N/A
Turfan et al., 2014 [54]	167	152	15	5.01 ± 3.25	4.83 ± 3	7.2 ± 4.8
Tasal et al., 2014 [55]	219	174	45	6.94 ± 6.26	6.1 ± 5.3	10.2 ± 8.4

The studies also showed that there is a strong association between high NLR values and mortality. The variability in mean and median NLR values across studies reflects differences in population characteristics and disease severity. There is heterogeneity in cutoff values, statistical measures, and population characteristics; the consistent association between elevated NLR and poor outcomes highlights its utility as a robust prognostic marker in HF patients.

**Figure 3:**
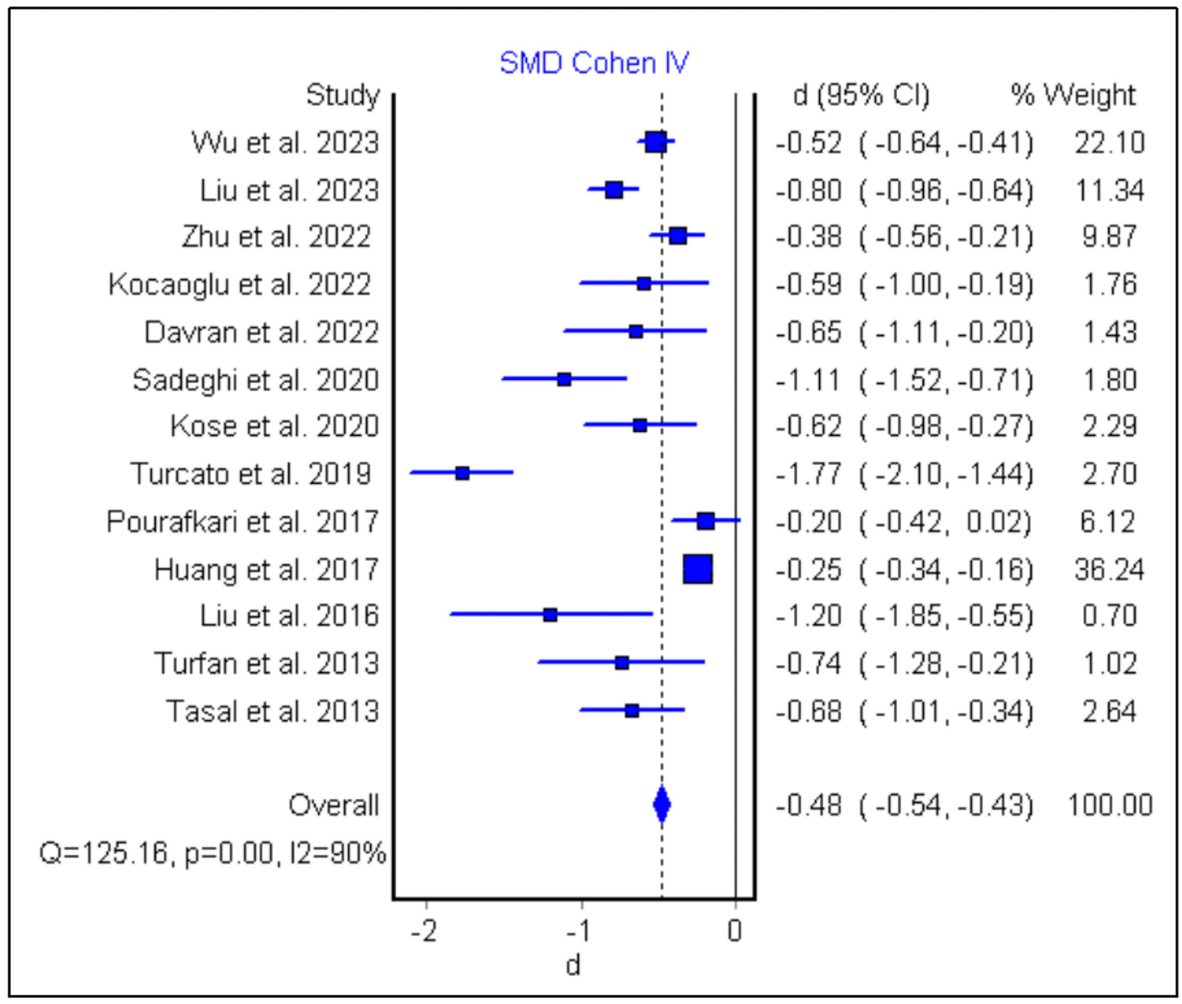
Forest plot showing the NLR values between survived HF patients and dead HF cases Wu et al., 2023 [20]; Liu et al., 2023 [22]; Zhu et al., 2022 [23]; Kocaoglu and Kocaoglu, 2022 [28]; Davran et al., 2022 [30]; Sadeghi et al., 2020 [37]; Kose et al., 2020 [38]; Turcato et al., 2019 [39]; Pourafkari et al., 2017 [44]; Huang et al., 2017 [14]; Liu et al., 2016 [47]; Turfan et al., 2014 [54]; Tasal et al., 2014 [55] NLR, neutrophil-to-lymphocyte ratio; HF, heart failure; CI, confidence interval; SMD, standardized mean difference

The forest plot (Figure 3) illustrates the standardized mean difference (SMD) of neutrophil-to-lymphocyte ratio (NLR) values between survived and deceased heart failure (HF) patients across multiple studies. Wu et al. (2023) have the highest weight (22.10%), indicating its substantial influence on the pooled results [20]. The pooled effect size, represented by the diamond at the bottom, is -0.48 (95% CI: -0.54, -0.43), demonstrating a statistically significant overall lower NLR in survived HF patients compared to deceased cases. However, the heterogeneity statistics (Q = 125.16; p = 0.00; I² = 90%) indicate high variability among the included studies, likely due to differences in study design, populations, or measurement methods. Overall, the plot highlights a significant association between elevated NLR and mortality in HF patients, despite the observed heterogeneity.

**Figure 4:**
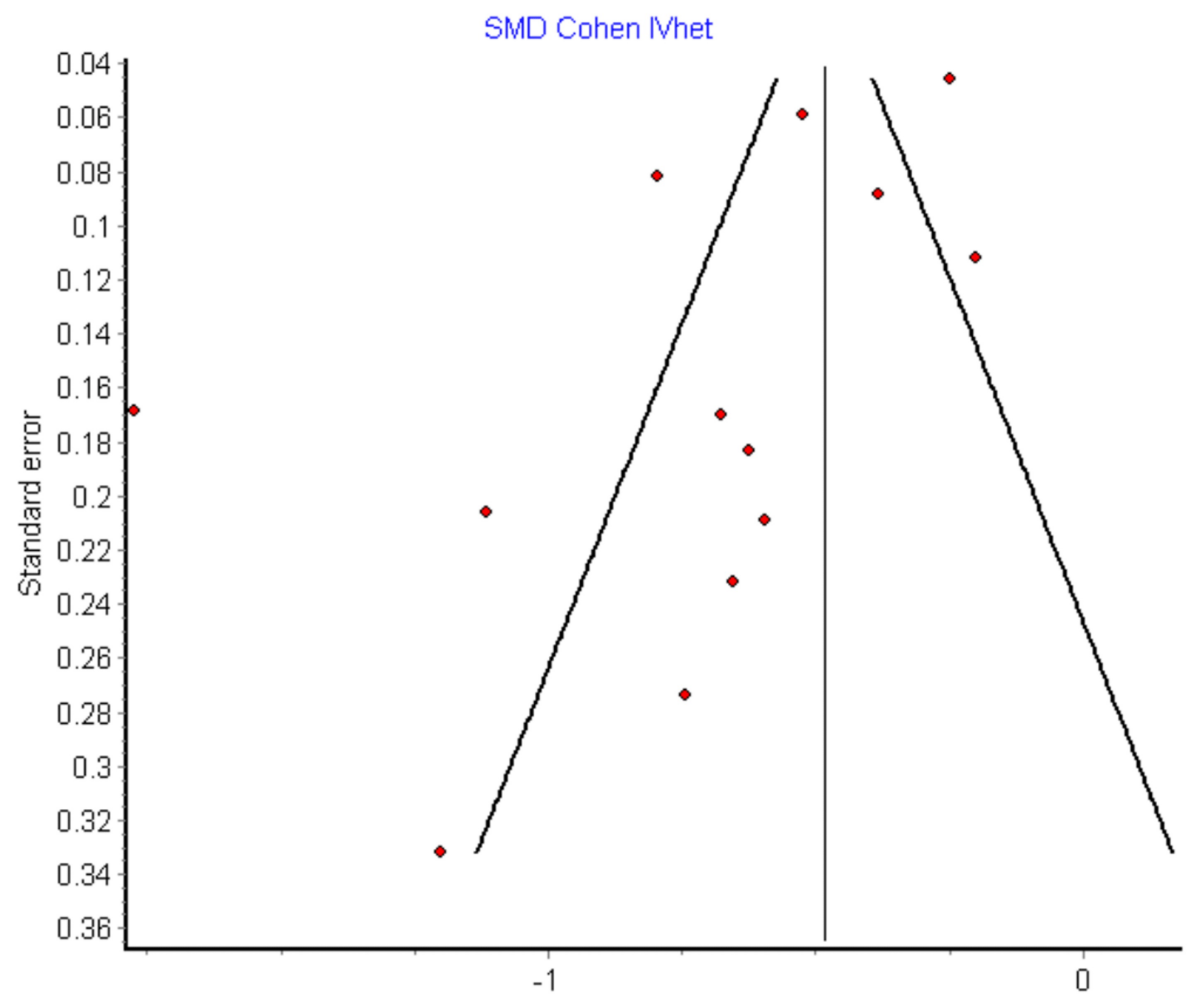
Funnel plot showing the NLR values between survived HF patients and dead HF cases NLR, neutrophil-to-lymphocyte ratio; HF, heart failure; SMD, standardized mean difference

Figure 4 shows the funnel plot of the relationship between the standardized mean difference (SMD) of neutrophil-to-lymphocyte ratio (NLR) values and their standard errors across studies, assessing potential publication bias in the analysis of survived versus deceased heart failure (HF) patients.

Table 3 presents the standardized mean differences (SMDs) and corresponding odds ratios (ORs) for each study, along with their 95% confidence intervals (CIs). Negative SMD values indicate that the neutrophil-to-lymphocyte ratio (NLR) is higher in patients who died compared to those who survived, suggesting an association between elevated NLR and increased mortality risk. The odds ratios further quantify this relationship, with OR values of less than 1 consistently indicating that higher NLR is associated with lower odds of survival. The study found a consistent and significant association between elevated neutrophil-to-lymphocyte ratio (NLR) values and increased mortality risk in heart failure (HF) patients. Standardized mean differences (SMDs) across studies are uniformly negative, indicating that NLR values are higher in deceased patients compared to survivors. Notably, studies such as by Turcato et al. (2019) [39], Sadeghi et al. (2020) [37], and Liu et al. (2016) [47] report large SMDs of -1.77, -1.11, and -1.2, respectively, reflecting a strong discriminatory capacity of NLR between outcome groups. The corresponding odds ratios (ORs) are all below 1, further reinforcing that higher NLR is associated with a decreased likelihood of survival. For example, Turcato et al. (2019) [39] report an OR of 0.377, suggesting a 62.3% reduction in survival odds with elevated NLR, while Sadeghi et al. (2020) [37] and Liu et al. (2016) [47] show ORs of 0.542 and 0.516, respectively, pointing to similar trends. Even studies with more moderate SMDs, such as by Wu et al. (2023) [20] and Zhu et al. (2022) [23], maintain consistent associations with ORs of 0.75 and 0.81, respectively. Although Pourafkari et al. (2017) report an SMD of -0.2 with a confidence interval crossing 0 and an OR of 0.895, suggesting a weaker and potentially nonsignificant effect, the overall body of evidence affirms that elevated NLR is a reliable and significant marker associated with mortality in HF patients [44]. This reinforces its potential clinical value in risk stratification and prognostication.

**Table 3:** Findings of the OR SMD, standardized mean difference; CI, confidence interval; OR, odds ratio

Study	SMD	CI_Lower	CI_Upper	OR	OR_CI_Lower	OR_CI_Upper
Wu et al., 2023 [20]	-0.52	-0.64	-0.41	0.750872061	0.702830167	0.7977899
Liu et al., 2023 [22]	-0.8	-0.96	-0.64	0.643521398	0.589217437	0.702830167
Zhu et al., 2022 [23]	-0.38	-0.56	-0.21	0.811086964	0.734503881	0.890733499
Kocaoglu and Alatli, 2022 [28]	-0.59	-0.79	-0.39	0.722462329	0.647076988	0.806630165
Davran et al., 2022 [30]	-0.65	-1.11	-0.19	0.698968222	0.542476778	0.900603666
Sadeghi et al., 2020 [37]	-1.11	-1.52	-0.71	0.542476778	0.432782494	0.676238131
Köse et al., 2020 [38]	-0.74	-1.11	-0.38	0.665151796	0.542476778	0.811086964
Turcato et al., 2019 [39]	-1.77	-2.1	-1.44	0.377090525	0.31439661	0.452286251
Pourafkari et al., 2017 [44]	-0.2	-0.42	0.02	0.895654986	0.793406165	1.011080944
Huang et al., 2017 [14]	-0.6	-0.82	-0.38	0.718492509	0.636468724	0.811086964
Liu et al., 2016 [47]	-1.2	-1.85	-0.55	0.516231485	0.360829403	0.738562167
Turfan et al., 2014 [54]	-0.74	-1.28	-0.34	0.665151796	0.493970243	0.829161773
Tasal et al., 2014 [55]	-0.68	-1.01	-0.34	0.687509247	0.573206066	0.829161773

The analysis of the ROC curve and the area under the curve (AUC) for the studies involving the neutrophil-to-lymphocyte ratio (NLR) demonstrates the effectiveness of NLR as a discriminatory marker. The AUC value of 0.834 indicates good discriminatory ability, meaning the model has an 83.4% chance of correctly distinguishing between positive and negative outcomes, such as survival versus mortality, based on NLR values. This suggests that NLR is a reliable predictor in the context of the study.

**Figure 5:**
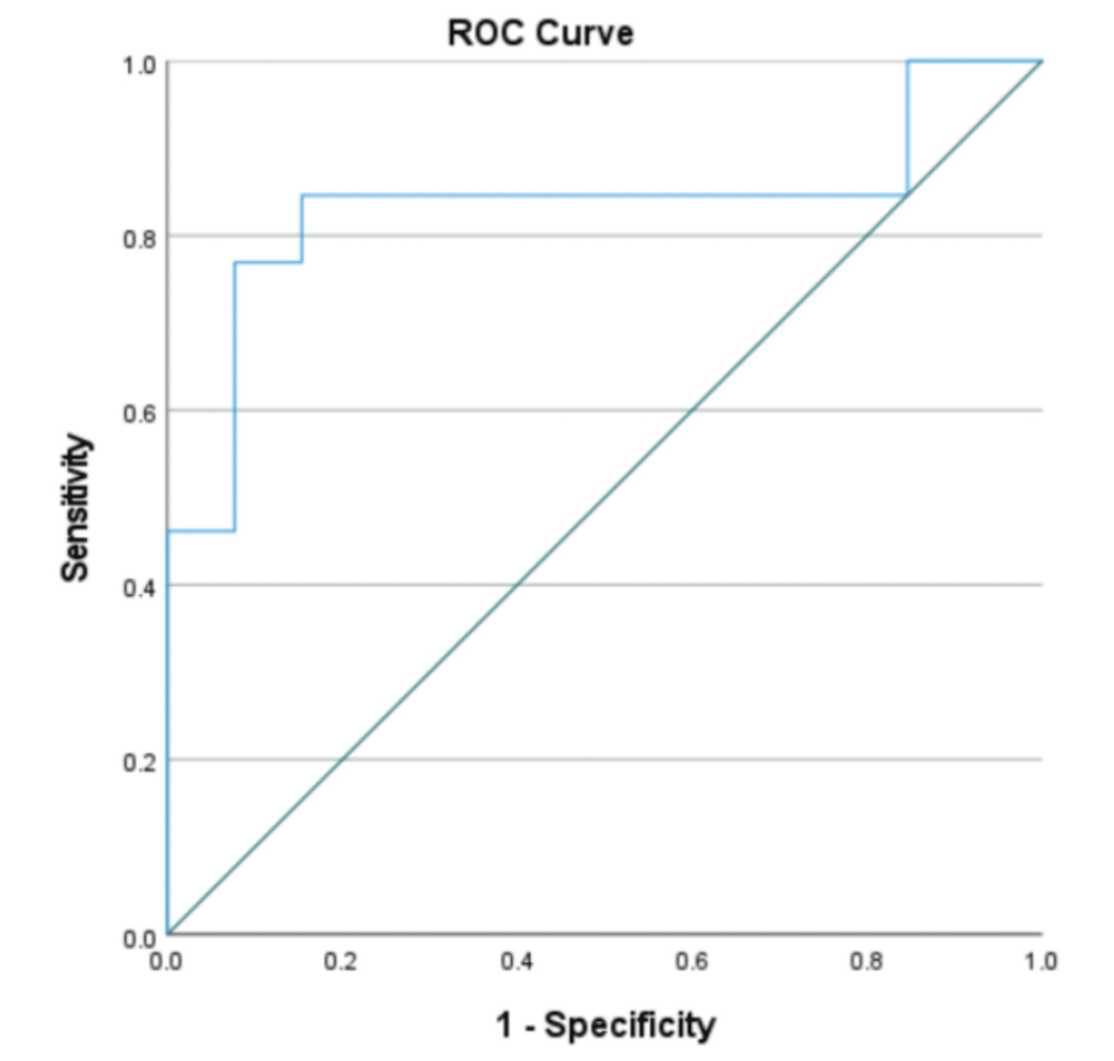
ROC showing the sensitivity and specificity of the NLR as found in these studies ROC, receiver operating characteristic; NLR, neutrophil-to-lymphocyte ratio

The best cutoff points were determined using Youden's index, which balances sensitivity and specificity. Among the evaluated cutoff values, 5.91 and 6.18 showed the highest Youden's index of 0.692. For the cutoff value of 5.91, sensitivity and specificity were both 84.6%, providing an optimal balance for accurate predictions. Similarly, at the cutoff value of 6.18, the sensitivity was 76.9% with the same level of specificity. These results highlight the usefulness of NLR in clinical decision-making, as these cutoff points can guide risk stratification and patient management strategies effectively (Figure 5).

Discussion

The current findings favor the relationship between raised NLR and increased morbidity and mortality in the heart failure population [9]. Published literature has shown that higher NLR is associated with poor prognosis in both acute and chronic heart failure. In a study among patients with acute decompensated heart failure, Cho et al. observed that patients having higher NLR had considerably higher rates of 30-day readmission and long-term mortality than those with lesser NLR [10]. Similarly, Benites-Zapata et al. [53] found that increased NLR was correlated with higher mortality or heart transplant risk in patients with advanced HF [1,6]. Mechanisms through which elevated NLR relates to poor outcomes in HF patients can be explained by the role of inflammation in the pathogenesis and progression of the disease. Neutrophils are the acute inflammatory response, whereas lymphocytes are part of the adaptive immune system and have been related to chronic inflammation. High NLR indicates that the innate immunity is dominant over the adaptive immune response, which implies increased inflammation [6,7]. This may further lead to the progression and deterioration of HF through various pathways. Neutrophil aggregation with platelets leads to the plugging of microvessels and results in myocardial ischemia (Table 4) [7].

**Table 4:** Key studies highlighting the prognostic value of neutrophil-to-lymphocyte ratio (NLR) in heart failure (HF) and related conditions LVAD, left ventricular assist device; CAD, coronary artery disease; CKD, chronic kidney disease

Key Findings	Clinical Implications	Citation
High NLR linked to increased mortality and right ventricular failure post-LVAD implantation	Highlights the importance of NLR in presurgical risk stratification for HF patients	[1,2]
Elevated NLR associated with adverse outcomes in elderly chronic HF patients	Indicates NLR as a prognostic marker for vulnerable HF populations, especially the elderly	[3]
High NLR correlated with higher 30-day readmission and long-term mortality in acute HF	Suggests using NLR to predict short- and long-term outcomes in acute HF patients	[4]
Elevated preoperative NLR predicts adverse events in non-cardiac surgeries after CAD	Suggests extending NLR usage for predicting outcomes in broader surgical populations	[5]
Increased NLR associated with worsening renal function in chronic HF	Demonstrates the role of NLR in monitoring renal complications in HF patients	[6]
High NLR predicts cardiovascular toxicity in high-risk cancer patients on immune checkpoint therapy	Highlights NLR's relevance beyond HF, particularly in cardio-oncology settings	[7]
NLR is a superior predictor of mortality in advanced HF compared to neutrophilia or lymphopenia	Reinforces NLR's value over traditional leukocyte counts in advanced HF management	[8]
High NLR increases all-cause mortality and cardiovascular events in CKD (systematic review)	Supports NLR as a universal marker of inflammation across cardiovascular and non-cardiovascular diseases	[9]

Lymphocytopenia has been associated with a high mortality rate and poorer prognosis in patients with severe HF [7]. Other inflammatory mediators, such as C-reactive protein, interleukin-6, and tumor necrosis factor-alpha, are also elevated in HF and contribute to the progression of the disease process [8]. The comprehensive nature of NLR, reflecting the balance between the innate and adaptive immune responses, makes it a valuable tool for the risk stratification and clinical management of HF patients. Several studies have shown that NLR is superior to independent neutrophil and lymphocyte counts in the prediction of mortality and poor outcomes in HF [6,10]. The neutrophil-to-lymphocyte ratio, therefore, is an expression of the balance between the innate and adaptive immune response and reflects the systemic inflammatory state in different stages of HF. The acute phase is the norm for the early stages of HF, typically marked by increased neutrophils as an indicator of innate immune system activation. This acute inflammatory state is most induced by myocardial injury, including ischemia or necrosis, and may be involved in the worsening of HF. With the progression of HF, the inflammation response tends to become chronic and is dominated by the adaptive immune system [50]. This manifests as a relative lymphopenia, with a subsequently increased NLR. The chronic inflammatory state is associated with the activation of various inflammatory mediators, such as cytokines and chemokines, which can further exacerbate myocardial dysfunction and remodeling. In advanced stages of HF, the imbalance between the innate and adaptive immune responses becomes more pronounced, with a marked increase in NLR. This reflects a predominance of the innate immune response, characterized by persistent neutrophilia and lymphocytopenia [10,48]. This chronic inflammatory status is associated with adverse events, such as the increased risk of mortality and heart transplant. The prognostic ability of NLR in HF has been well established since it has a comprehensive evaluation of the patient's inflammatory status. Increased NLR values reflect that there is a shift toward a pro-inflammatory status with a higher risk of an adverse event, including a risk of hospitalization and progression of disease and eventually death [13,50].

The strong statistical association observed between elevated NLR and increased mortality in heart failure patients underscores the potential clinical relevance of this biomarker. While the current meta-analysis aimed to provide a broad overview across diverse populations, we acknowledge that further stratification by ethnicity, age groups, and clinical settings could offer deeper insight into the generalizability of our findings. Although subgroup analysis was limited by inconsistent reporting of demographic-specific outcomes across the included studies, the consistent elevation of pooled NLR values in deceased patients suggests a prognostic role that warrants further investigation. The identified NLR thresholds (e.g., 5.91 and 6.18) may serve as preliminary reference points; however, we recognize that their optimal clinical applicability may vary by heart failure subtype and population. Future prospective studies that incorporate demographic stratification and standardized NLR cutoffs could significantly strengthen the translational value of this biomarker in routine clinical decision-making.

## Conclusions

The study has concluded that an elevated neutrophil-to-lymphocyte ratio (NLR) is consistently associated with increased mortality in patients with heart failure (HF). Across multiple datasets, deceased patients exhibited significantly higher NLR values compared to those who survived, and this trend was supported by the included studies' analyses. The study found a statistically significant standardized mean difference favoring higher NLR in non-survivors. ROC analysis further validated NLR's discriminative capability, with an area under the curve (AUC) of 0.834, indicating strong predictive performance. The strength of NLR, operating in different age groups, genders, and severities of HF, makes it a very adaptable marker of risk stratification and management.

The incorporation of NLR into routine clinical practice will enable doctors to better determine high-risk patients, personalize treatment strategies, and, in the future, improve outcomes during therapy in both acute and chronic scenarios of heart failure. Future studies should delve into establishing cutoffs for NLR and the predictive values that work alongside other biomarkers to improve the efficiency of guiding the therapeutic decision-making process.

## Appendices

Table 5 shows the search strategy and related details. 

**Table 5:** Search strings and related details

Search Strategy Component	Details
Databases used	PubMed, Scopus, Embase, and Web of Science
Publication coverage	Up to July 2023
Search fields in databases	Titles, abstracts, and keywords in Scopus; titles and abstracts in PubMed, Embase, and Web of Science
Search terms used	Combination of Medical Subject Headings (MeSH) and non-MeSH terms
Neutrophil-to-lymphocyte ratio (NLR) search terms	"neutrophil* to lymphocyte* ratio" OR "neutrophil-lymphocyte" OR "neutrophil*-lymphocyte* ratio" OR "neutrophil* to lymphocyte*" OR "neutrophil*-to-lymphocyte* ratio" OR "neutrophil*-to lymphocyte* ratio" OR "neutrophil* to-lymphocyte* ratio" OR "neutrophil/lymphocyte ratio" OR "neutrophil*/lymphocyte*" OR "nlr"
Heart failure (HF) search terms	"heart failure" OR "cardiac failure" OR "heart insufficiency" OR "cardiac insufficiency" OR "congestive heart failure" OR "congestive cardiac failure" OR "decompensated heart failure" OR "decompensated cardiac failure" OR "decompensated heart insufficiency" OR "decompensated cardiac insufficiency" OR "acute decompensated heart failure" OR "acute decompensated cardiac failure" OR "acute decompensated heart insufficiency" OR "acute decompensated cardiac insufficiency" OR "hf"
